# Highly Transparent and Bifacial Dye-Sensitized Solar Cells via Slot-Die Coating for Greenhouse-Integrated Agrivoltaics

**DOI:** 10.3390/ijms27157056

**Published:** 2026-08-06

**Authors:** Archontoula Nikolakopoulou, Dimitris A. Chalkias, Konstantinos C. Andrikopoulos, Dimitris F. Sampsonas, Aikaterini K. Andreopoulou, Elias Stathatos

**Affiliations:** 1 Nanotechnology & Advanced Materials Laboratory, Department of Electrical and Computer Engineering, University of the Peloponnese, GR26334 Patras, Greece; 2 Brite Hellas S.A., Industrial Zone of Patras, GR25018 Patras, Greece; 3 Department of Chemistry, University of Patras, GR26500 Rio-Patras, Greece

**Keywords:** dye-sensitized solar cell, slot-die coating, transparent, bifacial, spectral engineering, greenhouse compatibility factor, agrivoltaic performance factor

## Abstract

It is well-known nowadays that the usage of conventional opaque photovoltaics in agricultural practices has negative effects on crops growth, mainly due to the shading effect they cause. On the other hand, most of the emerging semi-transparent solar cells do not demonstrate the appropriate optical characteristics and scalability to attain their viable integration in agriculture, undermining their commercialization. This study deals with the development of wavelength-selective semi-transparent dye-sensitized solar cells (DSSCs) using the scalable slot-die deposition method. These devices are designed to provide high transparency in the photosynthetically active radiation (PAR) region and effectively exploit the near-ultraviolet to blue-visible spectrum for power production, simultaneously protecting cultivations from harmful short-wavelength irradiation. To this aim, a new quinoline-based dye and a highly transparent iodine-free electrolyte were employed in DSSCs, giving an external quantum efficiency of 70% for wavelengths up to 500 nm and a PAR transmittance on the level of 50% (55% crop growth factor). Additionally, the light-to-electricity conversion efficiency of these devices is high for both front- and rear-side illumination under all-weather irradiation conditions (up to 94% bifaciality factor). Finally, two new figures-of-merit (greenhouse compatibility factor, agrivoltaic performance factor) are introduced to quantify the balance of photovoltaic performance and agronomic functionality.

## 1. Introduction

Human overpopulation has led to increased water, energy and food consumption. This situation imposes significant pressure on meeting sustainability on our planet, especially in the domain of agriculture [1,2]. Consequently, the transition from conventional energy sources to renewable ones has become imperative, with solar energy being a clean and abundant alternative source of energy [3,4]. Based on the aforementioned, agrivoltaics have been introduced in the last few years as an effective strategy for combining agricultural practices with energy production on the same plot of land, thus greatly enhancing land-use efficiency. The adoption of agrivoltaics has demonstrated significant benefits in the field, such as increased clean energy production, stability of crops yield across all seasons, reduced irrigation requirements, and most importantly, a reduction in competition for land usage for energy and crop production [5,6]. However, the effective integration of solar energy conversion devices in agriculture mainly depends on the compatibility of the photovoltaic (PV) characteristics with existing agricultural practices, with market-available technologies not meeting the corresponding requirements adequately [7,8].

The selection of the PV technology for agrivoltaic systems has a direct impact on both energy and crop production. Conventional silicon-based PVs remain the most widely used in terrestrial applications, due to their high efficiency, prolonged stability and technological maturity. However, these solar cells are opaque, which is a highly critical characteristic that reduces and distorts the light reaching plants, thereby limiting the ability to simultaneously produce energy and crops. To deal with this challenge, the development of emerging PV technologies, such as dye-sensitized solar cells (DSSCs), organic solar cells and perovskite solar cells, has led to a significant step forward, especially in the transparency factor. Thanks to their wide range of design capabilities, these technologies can be seamlessly integrated into any application [9,10]. However, to do so and demonstrate commercial viability, their development through scalable deposition techniques is also necessary. Industrial-compatible methods, such as blade coating, screen-printing, slot-die coating and inkjet printing, are promising candidates for their upscaling [11]. Using printing techniques, the production of uniform and high-quality thin films, and correspondingly highly efficient solar cell devices on a large scale, has already been demonstrated for various solar cell technologies in the literature. Depending on the requirements of the devices design, different techniques are proposed, among which slot-die coating is a viable deposition method to attain high throughput and accuracy, at a low manufacturing cost [12].

Focused on agricultural applications, current research has highlighted the importance of spectral engineering of solar cells to ensure that energy production is balanced with the lighting and thermal comfort requirements for crops development in indoor and outdoor spaces. In agriculture, the transparency of the solar cells across the entire spectrum of photosynthetically active radiation (PAR), the intensity of UV radiation reaching the crops, as well as the light-to-electricity conversion efficiency under different lighting conditions, i.e., under diffuse or low light, are crucial to investigate [13]. Among the emerging PV technologies, DSSCs stand out as a particularly suitable technology for applications in agriculture. Except for their simple structure and low manufacturing cost, the excellent optical adaptability through the appropriate selection of solar cell materials that they offer is highly important [14].

To exploit the multiple advantages of DSSCs and fully utilize this technology in agricultural practices, various approaches have been proposed, with the majority of efforts being focused on the development of new dye-sensitizers, especially on Donor–π–Acceptor (D–π–A) systems. These systems allow precise spectral tuning to maximize light utilization, while maintaining plant-compatible transparency. Common donor units include triphenylamine, carbazole, indoline and phenothiazine, combined with appropriate π-spacers and anchoring acceptor groups (e.g., cyanoacrylic acid, carboxylic acid, catechol, etc.) to ensure efficient electron injection into TiO_2_ [15,16,17]. Recent studies have also focused on studying the growth, flowering and morphological response of plants to light conditions in greenhouses employing semi-transparent DSSCs as glazing materials [18,19]. In the direction of attaining highly transparent solar cell devices, the fabrication of colorless DSSCs and the employment of UV-selective organic absorbers have attracted attention in order to be used as cladding materials for cultivation underneath [20,21]. When these efforts are combined with strategies like the employment of transparent electrolytes and polymorphic TiO_2_ as light-scattering layers for solar cells that improve the spectral transmission and thermal stability of DSSCs, this technology is further supported for its integration in agrivoltaics practices [15,22]. These efforts have recorded significant values in calculations and measurements of optical and functional parameters for greenhouses. At the same time, the maturation of DSSCs technology using scalable manufacturing has been increasingly studied in recent years. However, although a number of efforts have been made to improve large-area devices fabrication, the printing of modules specifically designed for greenhouse integration is limited [23,24]. In order to address this gap and enable the integration of DSSCs into greenhouses, there is a need for further research into scalable methods. This would result in large-scale nano-PVs that could achieve the required spectral control of light for the simultaneous production of energy and crops.

This study presents the first report on the development of wavelength-selective DSSCs using the slot-die coating method, aiming at the fabrication of large-scale semi-transparent solar cells optimized for greenhouses integration. A newly designed UV-selective organic dye was employed to selectively absorb solar irradiation out of the PAR region, enabling the conversion of short-wavelength light into electrical power without hindering photosynthesis, while protecting the plants from harmful UV irradiation. A transparent-to-visible-light electrolyte was also tested in its application to the system to minimize absorption losses across the visible spectrum, increasing the device transparency and, consequently, the adaptability of the proposed technology in agricultural practices. A comprehensive evaluation of the optical and electrical characteristics of the solar cells was carried out, with particular emphasis on the spectral transmittance of the devices, taking into account the McCree action spectrum [25]. The bifacial functionality under all-weather irradiation conditions of the DSSCs was also evaluated, studying the ability of these solar cells to utilize incident light from all directions and intensities, determining their potential for real agricultural practices integration. Finally, two new agrivoltaic figures-of-merit are introduced to quantify the balance of PV performance and agronomic functionality.

## 2. Results and Discussion

**Figure 1:**
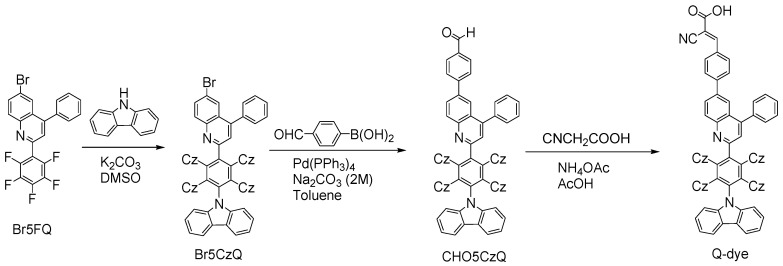
Synthetic steps for the quinoline-based dye.

To develop the spectral engineered DSSCs for greenhouse integration, a quinoline-based dye decorated with carbazole moieties was designed and synthesized to present a minimal overlap with the McCree action spectrum [25]. For this case, a quinoline electron acceptor core (Q) was used due to its high synthetic versatility, and favorable thermal and electrochemical stability, while carbazole (Cz) was chosen as a donor moiety, again considering its high thermal and electrochemical stability. The incorporation of strong electron donor and acceptor moieties ensures minimal overlap with the McCree action spectrum, while the large dihedral angles of the final dye molecule (between the carbazole and quinoline moieties) result in favorable energy levels. For the synthesis of the quinoline core (Br5FQ), a Friedlander synthesis was followed between 2-amino-5-bromobenzophenone and 1-(perfluorophenyl) ethanone [26]. Next, Br5FQ reacted with carbazole in DMSO, in the presence of K_2_CO_3_, to substitute all fluorine atoms with carbazole moieties. Then, the carbazole-substituted intermediate (Br5CzQ) reacted under palladium-mediated Suzuki coupling conditions with 4-formylphenylboronic acid, affording CHO5CzQ. Finally, the aldehyde functional group was converted to cyanoacrylic acid under Knoevenagel condensation conditions with 2-cyanoacetic acid, in the presence of NH_4_OAc in acetic acid. The synthetic steps for the dye molecule are presented in Figure 1. A detailed description of the synthetic procedure of the dye is given in the Supporting Information. For all syntheses, the success of the reactions was confirmed via ^1^H nuclear magnetic resonance (^1^H-NMR) in CDCl_3_ (Figure S1). The success of the carbazole substitution reaction was confirmed via ^19^F-NMR in CDCl_3_ (Figure S2).

**Figure 2:**
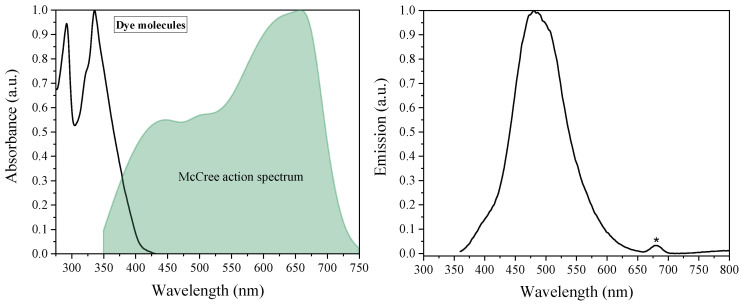
Absorbance (**left**) and emission (**right**) spectra of the dye in CHCl_3_ solution. The emission peak of the excitation laser in the emission spectrum is denoted with an asterisk (*).

The absorption and emission characteristics in solution of the synthesized dye were investigated in CHCl_3_, and presented in Figure 2. As it is observed, the dye has two absorption peaks, at 292 nm and 336 nm, ascribed to the π-π * transitions of the aromatic moieties of the dye. The absorption is far outside the absorption spectrum of the McCree action spectrum, confirming its suitability for greenhouse-integrated DSSCs applications [25]. The photoluminescence (PL) spectrum of the dye in CHCl_3_ was also investigated to probe the efficiency of the energy transfer from the donor to the acceptor units within the dye molecule. From the PL spectrum, it can be seen that the dye exhibits a single broad featureless emission band with a full width at half maximum (FWHM) of 96 nm. The profile of the peak can be ascribed to the large dihedral angle between the two chromophores (Cz and quinoline), which results in a higher-energy CT state. Since the donor carbazole molecules are connected to the quinoline acceptor core via σ bonds, the dihedral angles created between the donor and acceptor are not consistent or constant, creating this broad emission.

The energy levels of the dye were estimated using cyclic voltammetry. The dye was drop-cast from a CHCl_3_ solution on an fluorine-doped tin oxide (FTO) electrode to estimate the highest occupied molecular orbital (HOMO) and lowest unoccupied molecular orbital (LUMO) values in the solid state. Since the dye has four carbazole moieties (donor character) covalently linked to an acceptor quinoline core, the overall character of the dye is electron-donating. The HOMO energy level was calculated from the first oxidation onset potential using Equation (1) (Figure S3). The value of −5.2 eV in Equation (1) emerged from calibration of the Ag/AgCl electrode versus a normal hydrogen electrode (NHE) and considering that the NHE redox potential corresponds to −4.6 eV on the zero vacuum level scale [27,28,29]. $E_{ons}^{ox}$ is the onset determined for the oxidation peak in cyclic voltammetry (V) versus Ag/Ag^+^, while $E_{1/2}^{Fc}$ equals to (*E*_red_ + *E*_ox_)/2 vs. Ag/AgCl. The LUMO energy level was calculated using Equation (2), where the optical bandgap ($E_{g,opt}$) was calculated from the onset of the absorption spectrum in the thin film form (Equation (3)).

(1) $$E_{HOMO}=-e\;(E_{ons}^{ox}-E_{1/2}^{Fc})-5.2\;[\mathrm{eV}]$$

(2) $$E_{LUMO}=E_{g,opt}+E_{HOMO}\;[\mathrm{eV}]$$

(3) $$E_{g,opt}=\frac{1240}{\lambda_{onset}}$$

**Figure 3:**
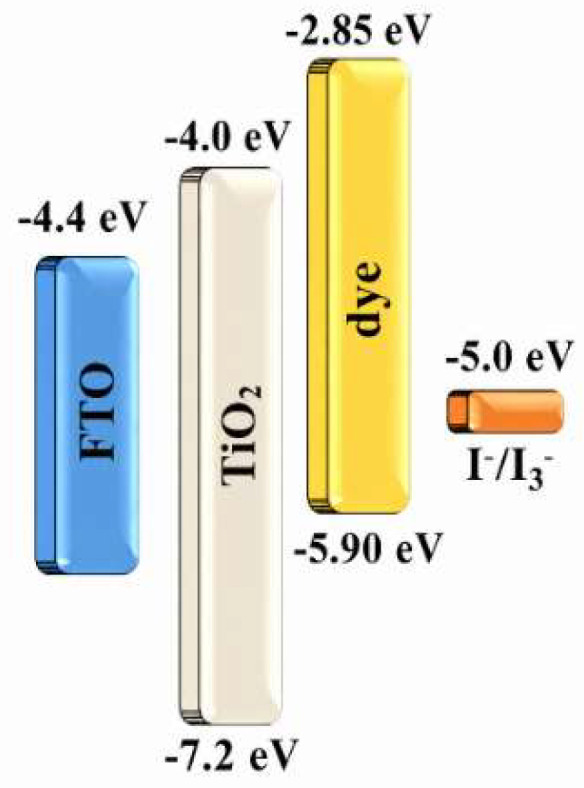
Schematic depiction of the energy levels of the quinoline-based dye compared to TiO_2_ and the I^−^/I_3_^−^ redox couple.

According to these calculations, the HOMO level of the dye was found to be −5.9 eV, which is deeper than the I^−^/I_3_^−^ redox couple; thus, the regeneration of the dye can take place. The LUMO level of the dye was estimated at −2.85 eV, providing a favorable energy scheme for electron injection from the dye to TiO_2_, as can be seen in Figure 3.

**Figure 4:**
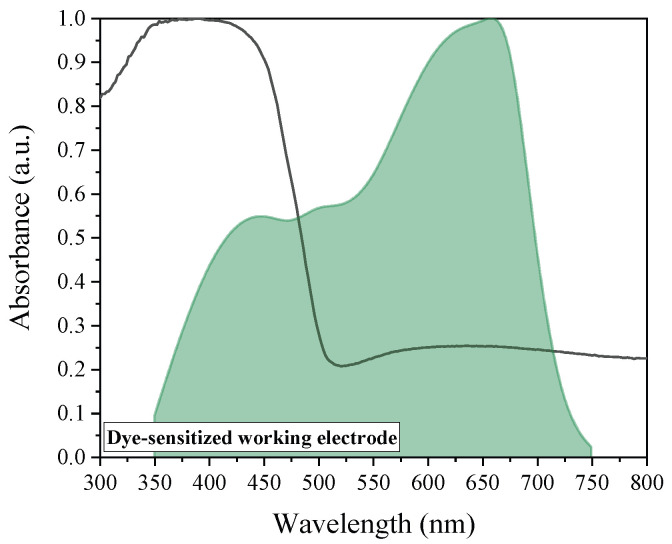
Ultraviolet-visible (UV-VIS) absorption spectrum of the quinoline-based dye-sensitized working electrode.

Figure 4 shows the ultraviolet-visible (UV-VIS) absorption spectrum of the dye-sensitized working electrode, namely the TiO_2_ scaffold on the FTO glass, sensitized by the quinoline-based dye. As evidenced, the system presents high light absorption up to about 500 nm, providing a high optical density for power production in the near-ultraviolet to blue-visible spectrum, and a high transparency for longer wavelengths, ensuring the transmittance of the light that is highly important for the photosynthetic action of the plants. Notably, there is a bathochromic shift when moving from the dye solution to the dye-sensitized TiO_2_ system. This shift predisposes a clustering of the dye molecules on TiO_2_ as J-aggregates, enabling a more intense and wider spectral range absorption of sunlight by the dye molecules, and correspondingly an increase in the photo-current production and light-to-electricity conversion efficiency of the solar cells [30].

**Figure 5:**
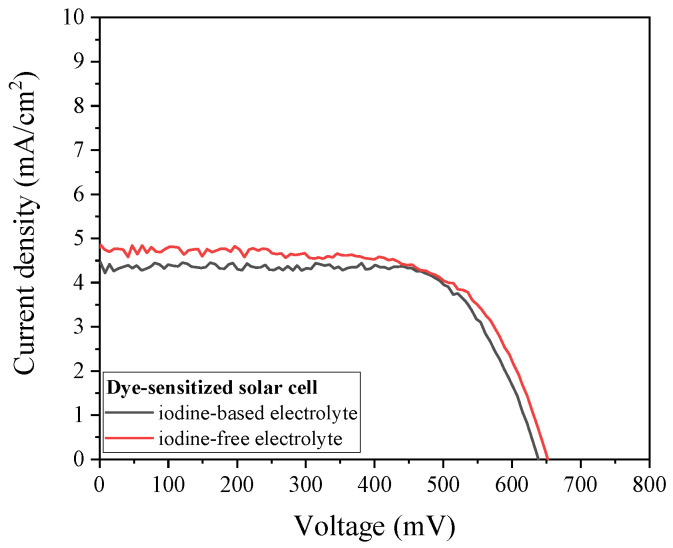
Current-density—Voltage (*J*–*V*) curves of the solar cells that employed the quinoline-based dye-sensitized working electrode and the iodine-based or iodine-free electrolyte.

Following the characterization of the synthesized dye and the dye-sensitized working electrode, small-sized (0.36 cm^2^) DSSC devices were fabricated and characterized. Figure 5 presents the characteristic current-density—voltage (*J*–*V*) curves of the solar cells employing either the conventional yellowish iodine-based electrolyte or the transparent-to-visible-light iodine-free electrolyte. The electrical characteristics of the solar cells, namely the short-circuit current density (*J_SC_*), open-circuit voltage (*V_OC_*), fill factor (*FF*) and power conversion efficiency (*PCE*), are tabulated in Table 1.

As evidenced, the DSSCs that employ the quinoline-based dye demonstrate *PCE* values on the order of 2%. Among them, the devices employing the iodine-free electrolyte demonstrate slightly higher values of *J_SC_* and *V_OC_* compared to the devices that employ the iodine-based electrolyte, thereby achieving a higher overall performance. On the other hand, the *FF* values of the former devices are lower compared to the latter devices. Similar reports are found in the literature [15,31]. Iodine-free electrolytes, such as the present one, are functional in DSSCs application since the I_3_^−^ species in these systems arise from the oxidation of the I^−^ species during the I^−^/I_3_^−^ redox reaction [32]. This ensures a minimum presence of I_3_^−^ species in the PV devices that absorb the visible light up to 450 nm and a minimum overlap with the absorption spectrum of the quinoline-based dye [33].

**Figure 6:**
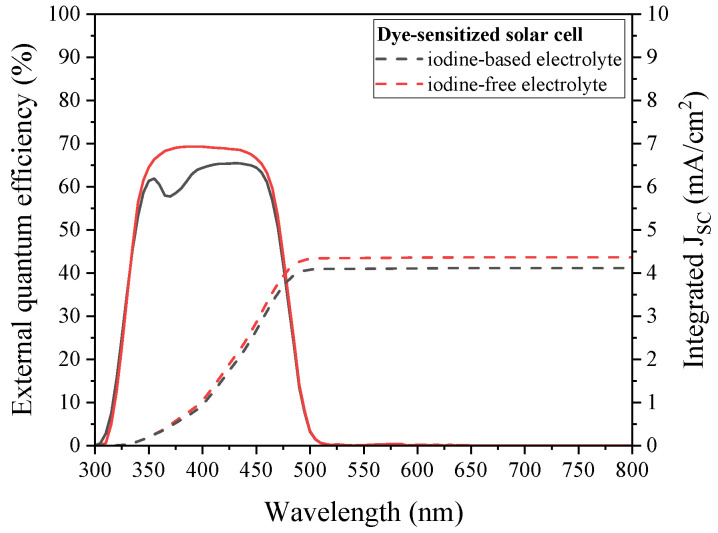
External quantum efficiency (EQE) of the solar cells that employed the quinoline-based dye-sensitized working electrode and the iodine-based or iodine-free electrolyte.

To give clearer evidence for the photo-current production by the solar cells, the external quantum efficiency (EQE) of the devices was measured and given in Figure 6. The integrated *J_SC_* values derived from the EQE were also calculated for comparison with the corresponding values obtained from the *J−V* characterization of the solar cells. As observed, the solar cells showed a high quantum efficiency response in the near-ultraviolet to blue-visible light spectrum, with the devices employing the iodine-free electrolyte demonstrating EQE values reaching the 70% between 350 and 460 nm. On the other hand, the devices that employ the iodine-based electrolyte demonstrated EQE values up to 65%, with a dip around 360 nm appearing, possibly due to the light absorption by the high number of intercalated triiodide species in the TiO_2_ scaffold, hindering light absorption by the dye-sensitizer [34]. At this point, it should be noted that the results arising from the *J–V* and EQE characterization of the solar cells are in agreement, with the values of *J_SC_* arising from the former characterization presenting an equal trend to the values arising from the latter characterization. The lower *J_SC_* values derived from the EQE can be ascribed to the existence of shallow traps in the solar cell materials, which can be filled under pre-illumination applied during the *J–V* measurements [35].

**Figure 7:**
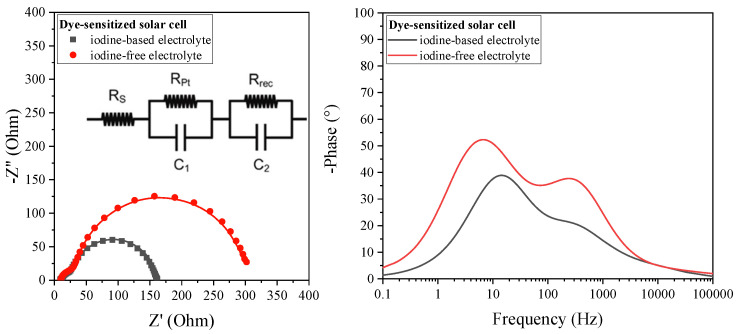
Nyquist plots (inset: the equivalent circuit) (**left**) and Bode phase diagrams (**right**) for the solar cells that employed the quinoline-based dye-sensitized working electrode and the iodine-based or iodine-free electrolyte.

In order to investigate the charge transfer kinetics inside the DSSCs, electrochemical impedance spectroscopy (EIS) measurements were carried out. By applying a small AC voltage perturbation to the DSSCs over a broad frequency range, distinct physicochemical processes with different characteristic time constants can be resolved, including charge transfer and recombination phenomena occurring at the various interfaces of the solar cells. Figure 7 shows the Nyquist plots and the Bode phase diagrams for the solar cells that employ the iodine-based or iodine-free electrolyte. The EIS parameters, namely the series resistance of the device (*R_S_*), the charge transfer resistance at the Pt/electrolyte interface (*R_Pt_*) and the charge recombination resistance at the TiO_2_/dye/electrolyte interface (*R_rec_*) obtained by fitting the experimental data (scatter symbols) with the equivalent circuit (lines) of the type *R_S_* (*R_Pt_*∥*C*_1_)(*R_rec_*∥*C*_2_) are tabulated in Table 2.

As it is observed, the Nyquist plots present two characteristic semicircles: the first semicircle, which is small in size, is presented at high frequencies regime, and corresponds to the charge transfer resistance at the Pt/electrolyte interface (*R_Pt_*); the second semicircle, which has a significantly larger diameter, appears at lower frequencies regime, and it is attributed to the charge recombination processes taking place at the TiO_2_/dye/electrolyte interface (*R_rec_*); the *R_S_* value of the solar cells is obtained from the ohmic resistance obtained by the Nyquist plot at the point where the curve intercepts with the real axis [36]. Considering the Bode phase plots, the frequency of the peaks observed in the middle-frequency domain can be used to evaluate the lifetime of electrons within the photo-anode (*τ_e_*), providing another indication of the charge recombination rate taking place at the TiO_2_/dye/electrolyte interface. By comparing the data, it is perceived that the usage of the iodine-free electrolyte in DSSCs leads to an increase in all *R_S_*, *R_Pt_*, *R_rec_* and *τ_e_* values. This suggests that the charges transfer inside the solar cells is partially inhibited by the usage of the iodine-free electrolyte compared to the iodine-based electrolyte, including the triiodide reduction to iodide at the Pt/electrolyte interface. This observation is attributed to the lower amount of triiodide species in the former electrolyte and correspondingly to its lower conductivity compared to the latter electrolyte [31]. On the other hand, the analysis suggests that the charge recombination rate near the TiO_2_/dye/electrolyte interface is reduced by the usage of the iodine-free electrolyte in DSSCs compared to the case of the iodine-based electrolyte. The variation of the EIS parameters supports the variation trend of the main electrical characteristics of the solar cells, where the *V_OC_* increases and the *FF* decreases for the devices that employed the iodine-free electrolyte.

**Figure 8:**
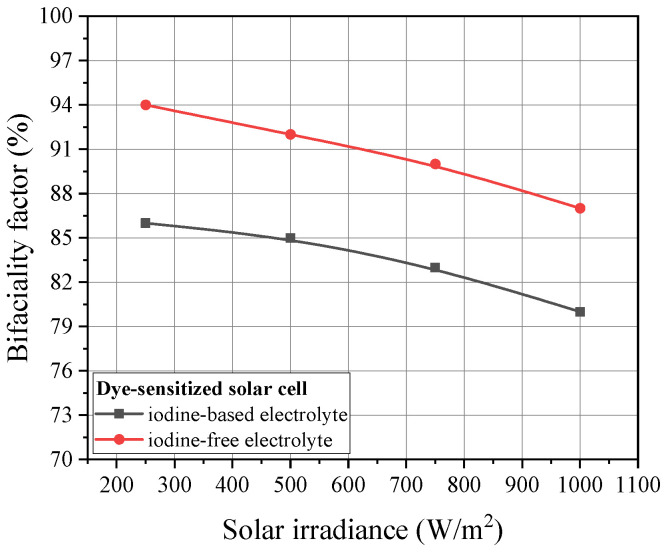
Bifaciality factor versus solar irradiance intensity for the solar cells that employed the quinoline-based dye-sensitized working electrode and the iodine-based or iodine-free electrolyte.

The bifaciality factor of the solar cells is also determined as a critical metric for devices that are intended to produce electricity from off-axis natural illumination. At this point, a diagram for the bifaciality factor versus solar irradiance intensity for the solar cells that employed the quinoline-based dye-sensitized working electrode and the iodine-based or iodine-free electrolyte is presented in Figure 8. Herein, the bifaciality factor is calculated as the ratio of the *PCE* attained by the illumination of the DSSC exclusively from the rear side to the corresponding value attained from the front side illumination [37].

According to this diagram, both types of DSSCs present a high bifaciality factor under all-weather irradiation conditions, which is of great importance for real-world agrivoltaic applications. By comparing the data, it is perceived that the solar cells that employ the iodine-free electrolyte present a higher bifaciality factor compared to the solar cells that employ the iodine-based electrolyte across the entire range of solar radiation intensities, reaching values up to 94%. This superior bifacial response originates from the reduced parasitic light absorption and improved optical transparency of the iodine-free electrolytic system, enabling more efficient rear-side photo-harvesting. It is known that the reduction or elimination of iodine-based redox species in DSSCs leads to improved performance under diffuse, low-intensity and indoor illumination conditions [38]. At this point, it is also noteworthy to mention that the reported values are among the highest records across other highly efficient bifacial emerging/commercialized solar cell technologies, according to current literature reports [39,40,41].

The evaluation of the impact of PV transparency on the plants growth is a highly important topic for consideration when designing spectral engineered solar cells for agricultural applications. For this case, crop growth factor (*G*) can be used as a metric to determine the ratio of the rate of the photosynthetic action of the plants under the greenhouse cladding material (herein, the DSSC) and the rate of the photosynthetic action of the plants under the full incident sunlight (without cladding material). *G* can be calculated using Equation (4), where *T* (λ) refers to the transmittance of the solar cell, *b_s_* (λ) is the AM 1.5 solar irradiance and *a* (λ) is the plant action spectrum [42]. Herein, the plant action spectrum is taken considering the McCREE curve [25].

(4) $$G=\frac{\int T\left(\lambda \right)b_{s}\left(\lambda \right)a\left(\lambda \right)d\lambda }{\int b_{s}\left(\lambda \right)a\left(\lambda \right)d\lambda }$$

**Figure 9:**
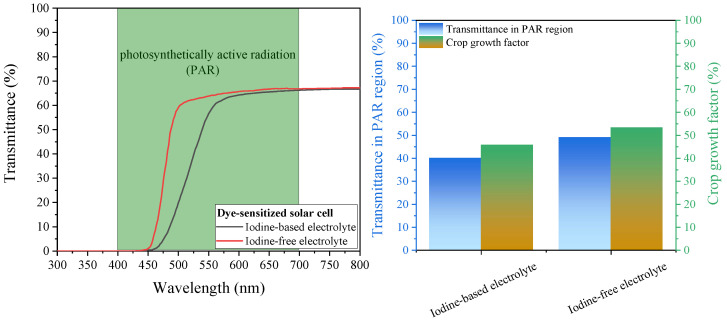
Transmittance spectra (**left**), transmittance in the photosynthetically active radiation (PAR) region and crop growth factor (**right**) for the solar cells that employed the quinoline-based dye-sensitized working electrode and the iodine-based or iodine-free electrolyte.

Figure 9 provides the transmittance spectra, the transmittance in the PAR region, along with the crop growth factor for the solar cells that employed the quinoline-based dye-sensitized working electrode and the iodine-based or iodine-free electrolyte. As evidenced, the spectral engineered solar cells demonstrate high transparency in their active area in the PAR region, and correspondingly high *G* values, which are determined to be up to 55%. When comparing the devices employing the iodine-based and iodine-free electrolyte, there is an increase in the values of the aforementioned metrics at the level of 15% for the devices employing the iodine-free electrolyte, attributed to the decreased amount of triiodide species that act detrimentally by absorbing light in the blue-green region of the solar spectrum. Noteworthily, the spectral transmission profile of these devices preferentially preserves green and red wavelengths, which contribute substantially to whole-canopy photosynthesis. Consequently, the proposed devices can be suitable for several greenhouse crops, particularly leafy vegetables and shade-tolerant species, highlighting the practical relevance of the proposed approach [43].

(5) $$GCF=\frac{G}{AVT}$$

(6) APF=PCE×G

**Figure 10:**
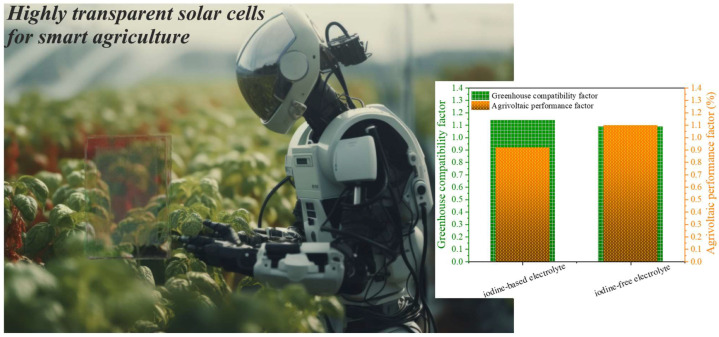
Greenhouse compatibility factor (*GCF*) and agrivoltaic performance factor (*APF*) for the solar cells that employed the quinoline-based dye-sensitized working electrode and the iodine-based or iodine-free electrolyte (the digital photo shows the high transparency of the spectral engineered solar cells; the background photo was taken from www.freepik.com, free license for personal and commercial purposes).

To evaluate the agronomic relevance, and the balance of PV performance and agronomic functionality, two new application-oriented figures-of-merit are introduced. The greenhouse compatibility factor (*GCF*) is introduced as an indication of the suitability of the transmitted light from cladding material (herein, the semi-transparent solar cell) for crop cultivation (see Equation (5)), where *G* is the crop growth factor as already defined, and *AVT* is the average visible transmittance that does not account for the wavelength-dependent photosynthetic effectiveness of the transmitted radiation. A *GCF* value higher than unity indicates that the transmitted spectrum from the cladding material is enriched in wavelengths that are particularly relevant for photosynthesis relative to the overall visible transmittance. Conversely, a *GCF* value lower than unity suggests that photosynthetically effective radiation is attenuated more strongly by the cladding material than implied by visible transparency alone. On the other hand, the agrivoltaic performance factor (*APF*) is introduced to quantify the simultaneous ability of a PV device to produce electricity and preserve photosynthetically effective radiation. Similar to the light utilization efficiency (*LUE*) in semi-transparent solar cells for building integration, *APF* can be determined by Equation (6), where *PCE* is the power conversion efficiency of the solar cell and *G* is the crop growth factor as already defined [44]. A higher *APF* indicates a more favorable balance between PV energy production and preservation of photosynthetically effective radiation. Figure 10 demonstrates the potential of the proposed slot-die-coated DSSC devices employing the quinoline-based dye-sensitized working electrode and the iodine-based or iodine-free electrolyte for the agricultural sector, as evaluated by *GCF* and *APF* metrics.

## 3. Materials and Methods

### 3.1. Materials

For the dye synthesis: 6-bromo-2-(perfluorophenyl)-4-phenylquinoline (Br5FQ) was synthesized according to literature procedures [26]. All solvents (sulfuric acid, acetic acid, toluene, dimethylformamide, chloroform, piperidine) and reagents (carbazole, potassium carbonate, sodium carbonate, 2′, 3′, 4′, 5′, 6′-Pentafluoroacetophenone, 4-formylphenylboronic acid, cyanoacetic acid) were purchased from Acros Organics (Geel, Belgium), Alfa Aesar (Heysham, UK), TCI (Tokyo, Japan), Fluorochem (Hadfield, UK) or Merck (Darmstadt, Germany), and used without further purification, unless otherwise stated. Palladium-tetrakis (triphenylphosphine) (Pd(PPh_3_)_4_) was recrystallized from absolute ethanol before use [45].

For the solar cell devices fabrication: FTO glass (Pilkington (Lancashire, UK), TEC 8, 3.85 mm thickness), titanium (di-isopropoxide) bis (2,4-pentanedionate) 75% in isopropanol (Alfa Aesar), titanium dioxide nano-powder (Degussa (Essen, Germany), P25), ethylene glycol monopropyl ether (Sigma Aldrich (St. Louis, MO, USA), assay 99.4%), titanium (IV) chloride (Sigma Aldrich, purity ≥ 99%), the synthesized dye, tetrahydrofuran (Fisher Chemical (Waltham, MA, USA), analytical reagent grade), 1-methyl-3-propylimidazolium iodide (Alfa Aesar, purity 98%), lithium iodide (Sigma Aldrich, purity 99.9%), iodine (Fisher Chemical, purity > 99.5%), 4-tertbutylpyridine (Sigma Aldrich, purity 96%), guanidine thiocyanate (Sigma Aldrich, purity ≥ 97%), acetonitrile (Merck, purity 99.9%), valeronitrile (Sigma Aldrich, purity 99.5%), dihydrogen hexachloroplatinate (IV) hexahydrate (Alfa Aesar, assay 99%), silver paste (Agar Scientific, Rotherham, UK) and low-temperature thermoplastic sealant (Greatcell Solar Materials (Queanbeyan East, Australia), 50 μm thickness) were used.

### 3.2. Fabrication of the Dye-Sensitized Solar Cells

The solar cells were developed under ambient atmosphere (20–25 °C and 40–60% RH) using a slot-die coater of the Ossila company (Sheffield, UK) equipped with a 50 mm head. More specifically, the working electrodes of the solar cells were based on a double-layered anode design, using an ultra-thin compact TiO_2_ film (blocking layer) and a thin mesoporous TiO_2_ film (scaffold). The blocking layer was used to prevent the back reaction of electrons with tri-iodide ions in the electrolyte [46]. It was developed by slot-die coating an ink composed of titanium (di-isopropoxide) bis (2, 4-pentanedionate) 75% in 2-propanol diluted 1/9 *v*/*v* in ethanol onto the FTO glass substrate. In this case, the ink was coated using the slot-die head employing a 50 μm thick shim for 25 mm coating width and a 100 μm thick meniscus guide with 500 μm lip depth for 25 mm coating width, at 300 μm coating gap; to develop the meniscus, the dispense rate and the time delay were set at 8 μL/s and 7 s, respectively, while the coating process was carried out using a speed of 15 mm/s and a dispense rate of 1 μL/s. Subsequently, the developed film was annealed at 500 °C for 10 min. The scaffold was developed on the top of the blocking layer by slot-die coating an ink prepared by a simple chemical technique, as reported in a previous work [47]. The formulation of the TiO_2_ paste was modified compared to the referenced recipe by its dilution (1:1 v:v) in ethylene glycol monopropyl ether to facilitate the paste slot-die coating. In this case, the ink was coated using the slot-die head employing a 200 μm thick shim for 25 mm coating width and a 100 μm thick meniscus guide with 500 μm lip depth for 25 mm coating width, at 300 μm coating gap; to develop the meniscus, the dispense rate and the time delay were set at 8 μL/s and 7 s, respectively, while the coating process was carried out using a speed of 15 mm/s and a dispense rate of 1 μL/s. The process was repeated nine times to attain the appropriate film thickness (about 15 μm). Subsequently, the developed film was sintered at 550 °C for 90 min, followed by gradual cooling to room temperature to avoid the development of cracking and peeling from the substrate. After the development of the scaffold, the working electrode was treated with TiCl_4_, in order to increase the surface area of the films for the dye sensitization [48]. This process was carried out by immersing the working electrode into a 40 mM TiCl_4_ aqueous solution at 70 °C for 30 min, followed again by sintering at 500 °C for 30 min and gradual cooling to room temperature. The sensitization of the working electrode was carried out overnight using a 0.3 mM dye solution, using tetrahydrofuran as a solvent. The counter electrodes were developed using platinum nanostructures that can attain high catalytic activity and transparency, simultaneously [49]. The nanostructured platinum thin film was developed by slot-die coating an ink composed of dihydrogen hexachloroplatinate (IV) hexahydrate (H_2_PtCl_6_(H_2_O)_6_) in 1-propanol (5 mg/mL). In this case, the ink was coated using the slot-die head employing a 50 μm thick shim for 25 mm coating width and a 100 μm thick meniscus guide with 500 μm lip depth for 25 mm coating width, at 300 μm coating gap; to develop the meniscus, the dispense rate and the time delay were set at 8 μL/s and 7 s, respectively, while the coating process was carried out using a speed of 15 mm/s and a dispense rate of 1 μL/s. The process was repeated five times to attain the appropriate film thickness. Subsequently, the developed film was annealed at 500 °C for 10 min. Finally, the fabrication of the solar cells was completed by sandwiching the dye-sensitized working electrodes with the counter electrodes, separated by a low-temperature thermoplastic spacer/sealant of 50 μm thickness. The sealing process was carried out at 120 °C for 15 min under constant pressure given by two clips. The stability of the dye is ensured at the sealing temperature employed, as evident from the thermogravimetric analysis (TGA) (Figure S4). The intervening space was filled using a liquid state iodine-based electrolyte (0.6 M 1-methyl-3-propylimidazolium iodide, 0.03 M iodine, 0.5 M 4-tertbutylpyridine and 0.1 M guanidine thiocyanate, in a mixture of acetonitrile and valeronitrile (volume ratio: 85:15)) or iodine-free electrolyte (0.6 M 1-methyl-3-propylimidazolium iodide, 0.1 M lithium iodide, 0.5 M 4-tertbutylpyridine and 0.1 M guanidine thiocyanate, in a mixture of acetonitrile and valeronitrile (volume ratio: 85:15)), using vacuum injection process through a hole developed to the counter electrode. The fabrication process of the solar cells was completed by sealing this hole using an extra piece of glass, under the same sealing conditions. The contacts of the solar cells were made of silver, using Electrodag 1415 silver paste, to collect the current efficiently. In all cases, the active area of the solar cells was equal to 0.36 cm^2^, equal to a proper shading mask that covered the rest of the electrode, preventing light incidence from the edges.

### 3.3. Characterization Methods

^1^H NMR spectra were recorded on a Bruker Advance DPX 400 MHz spectrometer (Bruker BioSpin GmbH, Magnet Division, Karlsruhe, Germany) in deuterated chloroform (CDCl_3_, 99.80 atom% Deutero DE) with TMS as internal standard. The UV-VIS spectra were recorded using a Hitachi—Science and Technology—U-1800 spectrophotometer (Tokyo, Japan). PL spectra were recorded on a Perkin Elmer LS45 fluorescence spectrometer (Waltham, MA, USA), using a 330 nm excitation wavelength. Cyclic voltammetry was carried out on an Autolab PGSTAT302N Potentiostat (Metrohm Autolab B.V., Utrecht, The Netherlands) in HPLC-grade acetonitrile (CH_3_CN), using tetrabutylammonium hexafluorophosphate (TBAPF_6_) as supporting electrolyte at 0.1 M concentration, using a scan rate of 100 mV/s. The working electrode was an FTO glass substrate, and the counter electrode was a Pt wire. The reference electrode was an Ag/AgCl electrode, and the electrochemical pair of ferrocene/ferrocenium ion was used as a standard. The dye was drop-cast onto the working electrode and dried overnight at 80 °C. Before carrying out the measurements, the cell was purged with pure argon for 20 min to remove diluted gases. Diffuse reflectance spectroscopy (DRS) measurements on the dye-sensitized working electrodes were carried out on a Jasco V-770 spectrophotometer (Tokyo, Japan) equipped with a 60 mm integrating sphere, embedding a PbS detector (ISN-923), from 300 to 800 nm, using an interval wavelength of 1 nm. UV-VIS transmittance spectroscopy measurements on the solar cell devices were recorded on a Hitachi U-2900 spectrophotometer (Tokyo, Japan), from 300 to 800 nm, using an interval wavelength of 2 nm. The characterization of the solar cell devices was carried out under standard testing conditions (AM 1.5 G, 100 mW/cm^2^, 25 °C), using a solar simulator of the model Solar Light (16S-300) (Solar Light Company, Glenside, PA, USA), calibrated by a reference cell (Newport 919P-003-10) (Newport Corporation, Irvine, CA, USA). The *J–V* curves of the devices were recorded using a Keithley 2601 source meter (Cleveland, OH, USA). The EQE of the solar cells was measured on a ThetaMetrisis PM-QE system, which was equipped with a Xenon (Xe) light source, employing a filter monochromator (Oriel Cornerstone™ 260 1/4 m, Newport), which was controlled by the software PM-Monitor^®^ v1.0; the measurements were carried out from 300 nm to 800 nm, using an interval wavelength of 5 nm and a delay time of 0.2 s. The EIS measurements on the solar cell devices were carried out on a potentiostat/galvanostat (Model PGSTAT 128 N) (Metrohm Autolab B.V., Utrecht, The Netherlands), and the data were recorded at *−V_OC_* forward bias, using a perturbation of ±10 mV, over the frequency range from 100 kHz to 0.1 Hz, in the dark and at room temperature.

## 4. Conclusions

Greenhouse-integrated agrivoltaics represent key energy devices toward the modernization of agriculture, offering the potential to simultaneously produce renewable electricity, maintain high-yield crop cultivation within the same land area and reduce the agricultural practice footprint. Among the different approaches, semi-transparent bifacial solar cells with in-active-area transparency are especially appealing to overcome the challenges that emerge by direct-sunlight-operating module devices with patterned apertures. In the present work, a highly transparent and bifacial spectral engineered DSSC device is successfully developed using scalable slot-die coating method and proposed as a cladding material for greenhouses integration.

By applying a newly synthesized quinoline-based dye (developed using simple routes that allow high yields of materials), solar cells that harvest and convert the near-ultraviolet to blue-visible light spectrum into electricity are developed, providing high transparency in the PAR region, which is a prerequisite for the photosynthetic action of the plants. As an extra improvement, the conventional iodine-based electrolyte of these devices is replaced by a highly transparent-to-visible-light electrolyte, eliminating the parasitic absorption usually caused by triiodide species in these devices. As a result, slot-die-coated PV devices with quantum efficiency on the level of 70% in the 350 and 460 nm region (*PCE* 2%), high bifaciality factor (up to 94%) under all-weather irradiation intensities and transparency >60% for wavelengths higher than 500 nm (55% crop growth factor) are developed.

Finally, two new agrivoltaic figures-of-merit, namely “greenhouse compatibility factor” and “agrivoltaic performance factor”, are introduced to quantify the balance of PV performance and agronomic functionality. This study gives new insights into the field of energy-autonomous greenhouses, further proving the importance of spectral engineering as an effective strategy to improve the compatibility of solar energy conversion devices with niche applications.

## Figures and Tables

**Table 1:** Electrical characteristics of the solar cells that employed the quinoline-based dye-sensitized working electrode and the iodine-based or iodine-free electrolyte.

Solar Cell	*J_SC_* (mA/cm^2^)	*V_OC_* (mV)	*FF* (-)	*PCE* (%)
iodine-based electrolyte	4.5 ± 0.1	638 ± 4	0.70 ± 0.01	2.01 ± 0.00
iodine-free electrolyte	4.8 ± 0.2	652 ± 5	0.66 ± 0.02	2.06 ± 0.01

At least three identical devices were characterized to obtain the reported mean values and standard deviations.

**Table 2:** Parameters obtained from the electrochemical impedance spectroscopy (EIS) measurements for the solar cells that employed the quinoline-based dye-sensitized working electrode and the iodine-based or iodine-free electrolyte.

Solar Cell	*R_S_* (Ohm)	*R_Pt_* (Ohm)	*R_rec_* (Ohm)	*τ_e_* (ms)
iodine-based electrolyte	9.2	31	136	10.6
iodine-free electrolyte	9.8	42	288	22.7

Measurements were carried out at *V = V_OC_* in the dark.

## Data Availability

The original contributions presented in the study are included in the article/Supplementary Materials, further inquiries can be directed to the corresponding author.

## Supplementary Materials

The following supporting information can be downloaded at: https://www.mdpi.com/article/10.3390/ijms27157056/s1.
