# Supplementary material for: Highly Transparent and Bifacial Dye-Sensitized Solar Cells via Slot-Die Coating for Greenhouse-Integrated Agrivoltaics
Source: Int J Mol Sci. 2026 Aug 6;27(15):7056. doi: 10.3390/ijms27157056 (PMC13466979; doi:10.3390/ijms27157056)
Supplement: Supplementary file 1 [file ijms-27-07056-s001.zip › ijms-4426442-supplementary.pdf]

Highly Transparent and Bifacial Dye-Sensitized Solar Cells via Slot-Die Coating for Greenhouse-Integrated Agrivoltaics

Archontoula Nikolakopoulou [1], Dimitris A. Chalkias [1,2,*], Konstantinos C. Andrikopoulos [3], Dimitris F. Sampsonas [1], Aikaterini K. Andreopoulou [3,*] and Elias Stathatos [1]

[1] Nanotechnology & Advanced Materials Laboratory, Department of Electrical and Computer Engineering, University of the Peloponnese, GR26334 Patras, Greece

[2] Brite Hellas S.A., Industrial Zone of Patras, GR25018 Patras, Greece

[3] Department of Chemistry, University of Patras, GR26500 Rio-Patras, Greece

* Correspondence: chalkias@mech.upatras.gr (D.A.C.); andreopo@upatras.gr (A.K.A.)

Supplementary Information

Synthetic Route of the Quinoline-based Dye

6-bromo-2-(2,3,4,5,6- tetra(9H-carbazol-9-yl)phenyl)-4-phenylquinoline (Br5CzQ): In a flame-dried and Ar-purged round-bottom flask, Br5FQ (5 g, 0.011 mol), carbazole (11.140 g, 0.067 mol) and K_2CO_3 (15.350 g, 0.111 mol) were added. Then, 100 mL DMSO were added, and the reaction mixture was left at 120 °C for 24 hours under an Ar atmosphere. After the reaction was completed, the reaction slurry was cooled to room temperature and poured into deionized water. Next, the solid precipitate was filtered, and extensively washed with petroleum ether and methanol. The final product was dried under vacuum at 50 °C for 18 hours. Yield 85%.

^{1}H NMR (600 MHz, $CDCl_3$): δ (ppm) = 7.58-7.54 (m, 3H Q), 7.41-7.24 (three m, 20H Cz), 7.17-7.14 (t, 2H Q), 6.91- 6.67 (three m, 20H Cz), 6.64-6.61 (t, 1H Q), 6.48 (s, 1H Q) 6.31 (d, 2H Q)

^{13}C NMR (150 MHz, $CDCl_3$): δ (ppm) = 140.53, 139.63, 138.78, 138.71, 138.23, 137.25, 136.56, 128.71, 128.16, 128.09, 126.73, 125.44, 125.06, 124.64, 124.27, 124.22, 124.17, 123.97, 123.69, 123.55, 123.47, 123.14, 122.40, 119.94, 119.84, 119.56, 119.38, 119.26, 111.12, 110.86, 110.69, 110.07

Academic Editor: Qinhe Pan

Received: 24 June 2026
Revised: 27 July 2026
Accepted: 3 August 2026
Published: 6 August 2026

Copyright: © 2026 by the authors. Licensee MDPI, Basel, Switzerland. This article is an open access article distributed under the terms and conditions of the Creative Commons Attribution (CC BY) license.

Br5CzQ

^{1}H NMR ($CDCl_3$)

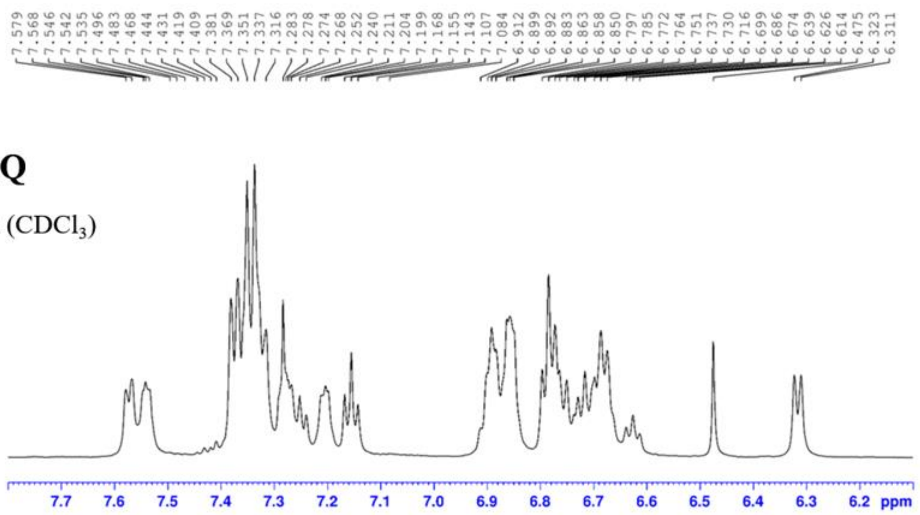

^{13}C NMR ($CDCl_3$)

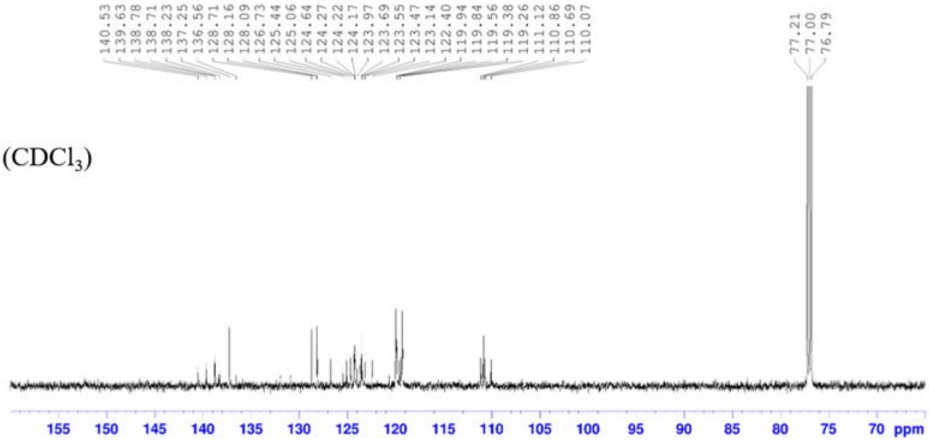

4-(2-(2,3,4,5,6-tetra(9H-carbazol-9-yl)phenyl)-4-phenylquinolin-6-yl)benzaldehyde (CHO5CzQ): In a flame-dried and Ar-purged round-bottom flask, Br5CzQ (1.055 g, 0.89 mmol), 4-formylphenylboronic acid (200 mg, 1.33 mmol), Na_2CO_3 (565.5 mg, 5.34 mmol) and $Pd(PPh_3)_4$ (31 mg, 0.027 mmol) were added. Then, 20 ml toluene were added, and the reaction mixture was left at 120 °C for 2 days under an Ar atmosphere. The reaction progress was evaluated with TLC in $CHCl_3$ eluent. After the reaction was completed, the slurry was filtered through a filter paper and then extracted with brine, $NaHCO_3$ (aq) and dH_2O, consecutively. Then, the organic phase was rotovaped to dryness and recrystallized from methanol. Lastly, the precipitate was filtered, washed extensively with methanol and dried under vacuum at 50 °C for 18 hours. Yield 65%.

^{1}H NMR (600 MHz, $CDCl_3$): δ (ppm) = 9.95 (s, 1H CHO), 7.80-7.78 (d, 2H ArCHO), 7.53-7.41 (m, 9H: 2H ArCHO - 3H Q - 4H Cz), 7.36-7.29 (m, 12H Cz), 7.26-7.21 (m, 4H Cz), 7.16-7.14 (t, 2H Q), 6.90-6.83 (m, 10H Cz), 6.77-6.75 and 6.69-6.67 (two m, 10H Cz), 6.61-6.59 (t, 1H Q), 6.52 (s, 1H Q), 6.37-6.36 (d, 2H Q).

^{13}C NMR (150 MHz, $CDCl_3$): δ (ppm) = 191.89, 140.68, 139.83, 138.91, 138.55, 138.39, 137.46, 130.27, 128.97, 128.63, 128.34, 127.85, 125.26, 124.83, 124.58, 124.49, 124.40, 124.13, 123.88, 123.71, 123.61, 123.32, 122.62, 120.17, 120.11, 120.07, 119.97, 119.71, 119.52, 119.39, 111.10, 111.02, 110.85, 110.28.

CHO5CzQ

^{1}H NMR ($CDCl_3$)

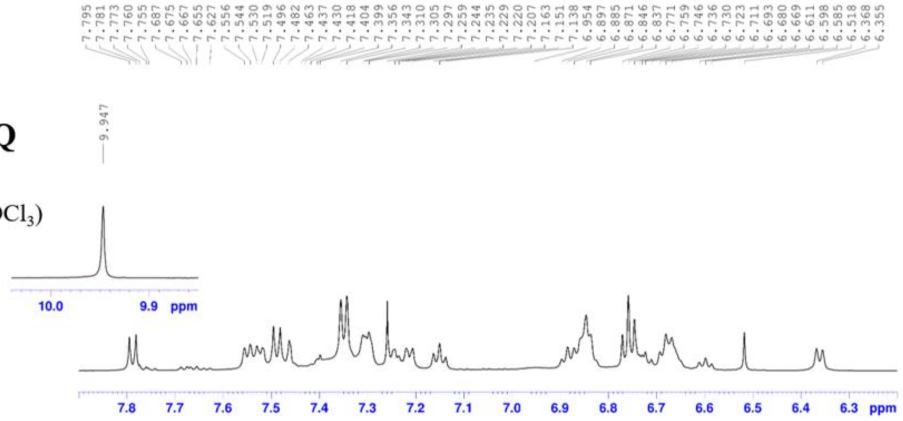

^{13}C NMR ($CDCl_3$)

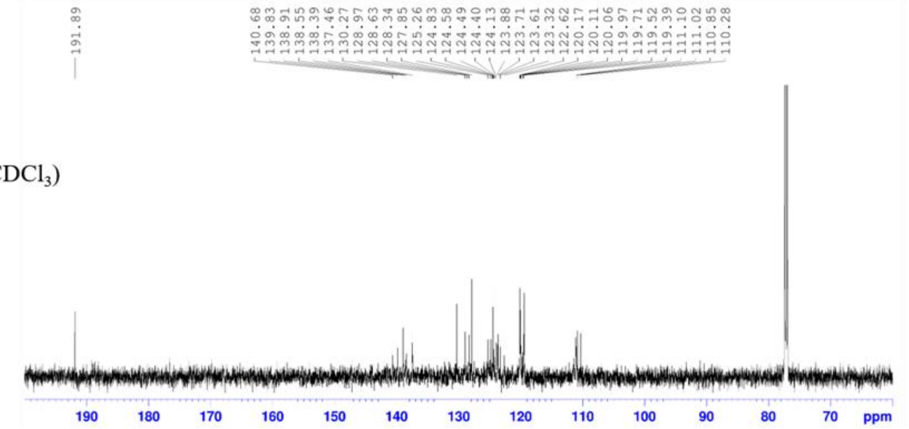

3-(4-(2-(4-(9H-carbazol-9-yl)phenyl)-4-phenylquinolin-6-yl)phenyl)-2-cyanoacrylic acid (5CzQ-CNacetic): In a flame-dried and Ar-purged round-bottom flask, CHO5CzQ (500 mg, 0.41 mmol), cyanoacetic acid (53 mg, 0.62 mmol) and NH_4OAc (32 mg, 0.42 mmol) were added. Then, 10 ml acetic acid were added, and the reaction mixture was left stirring under an Ar atmosphere at 140 °C for 1 day. The progress of the reaction was evaluated with TLC with $CHCl_3$ as an eluent. When the reaction was completed, the product was extracted using dH_2O and 20% aqueous HCl. The final product was rotovaped to dryness and washed extensively with methanol. Finally, the product was dried under vacuum at 50 °C for 18 hours. Yield 60%.

^1H NMR (600 MHz, $CDCl_3$): δ (ppm) = 8.29 (s, 1H CH=C), 7.99-7.98 (d, 2H Ar-CH=C), 7.56-7.41 (m, 9H: 2H Ar-CH=C - 3H Q - 4H Cz), 7.38-7.28 (m, 12H Cz), 7.24-7.13 (m, 4H Cz), 7.07-7.01 (m, 2H Q), 6.89-6.82 (m, 10H Cz), 6.78-6.62 (two m, 10H Cz), 6.61-6.57 (m, 1H Q), 6.52 (s, 1H Q), 6.39-6.38 (d, 2H Q).

^{13}C NMR (150 MHz, CD_3CN): δ (ppm) = 162.98, 154.48, 153.19, 143.79, 141.19, 140.40, 139.43, 139.05, 138.33, 138.29, 138.24, 137.53, 136.98, 132.05, 131.70, 129.04, 129.01, 128.98, 128.91, 128.76, 128.74, 128.69, 128.64, 128.57, 127.96, 127.09, 125.12, 125.03, 124.68, 124.58, 124.40, 123.54, 123.32, 123.23, 123.17, 123.07, 122.89, 122.12, 120.27, 120.21, 120.11, 119.69, 119.55, 119.48, 119.37, 119.33, 119.30, 119.23, 119.14, 118.29, 118.18, 112.45, 112.30, 112.15, 112.02, 111.69.

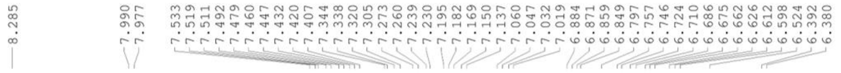

5CzQ-CNacetic

^{1}H NMR ($CDCl_3$)

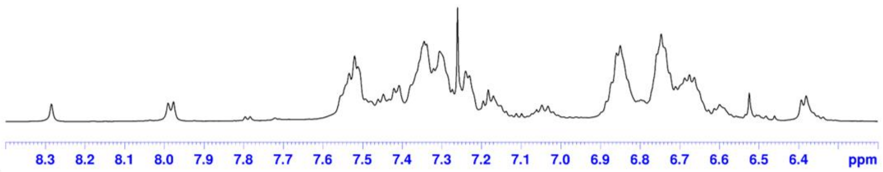

^{13}C NMR (CD_3CN)

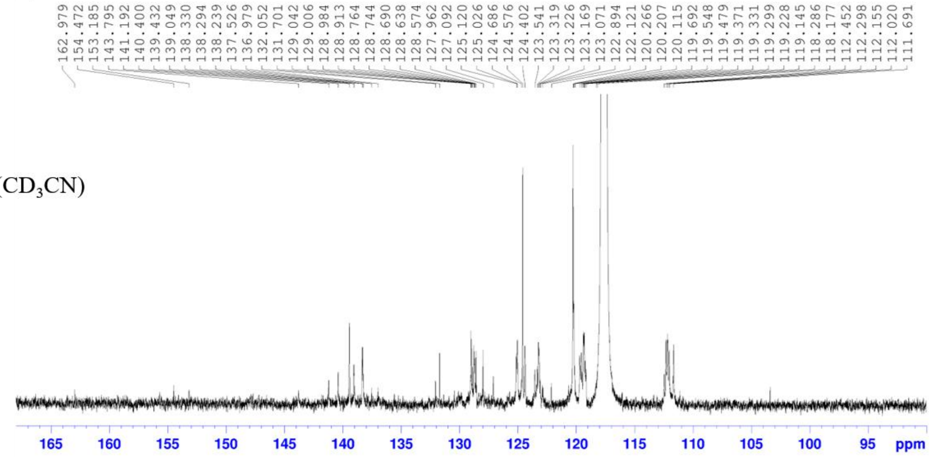

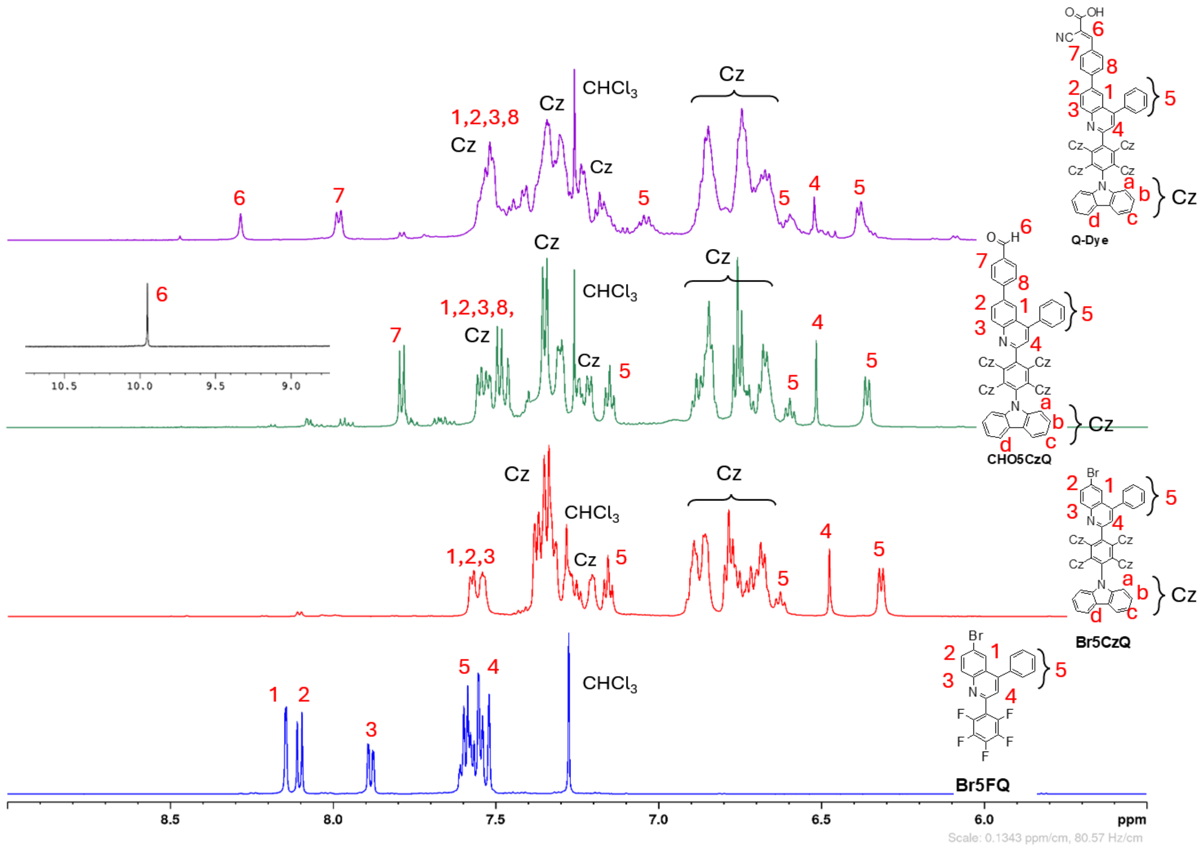

Figure S1. ^{1}H-NMR spectra of the SMMs synthesized in the study. Recorded in $CDCl_3$.

From the NMR spectra, the successful introduction of the carbazole moieties in the quinoline core is evidenced by the large shift of the peaks upfield, which is brought about by the shielding effect the electron-donating carbazole moieties have on the quinoline moiety. The introduction of the aldehyde (peak 6 of the black inset) and the cyanoacetic acid (peaks 6, 7 in the top magenta spectrum) functional groups can also be confirmed from the characteristic deshielded nature of the protons of the functional groups.

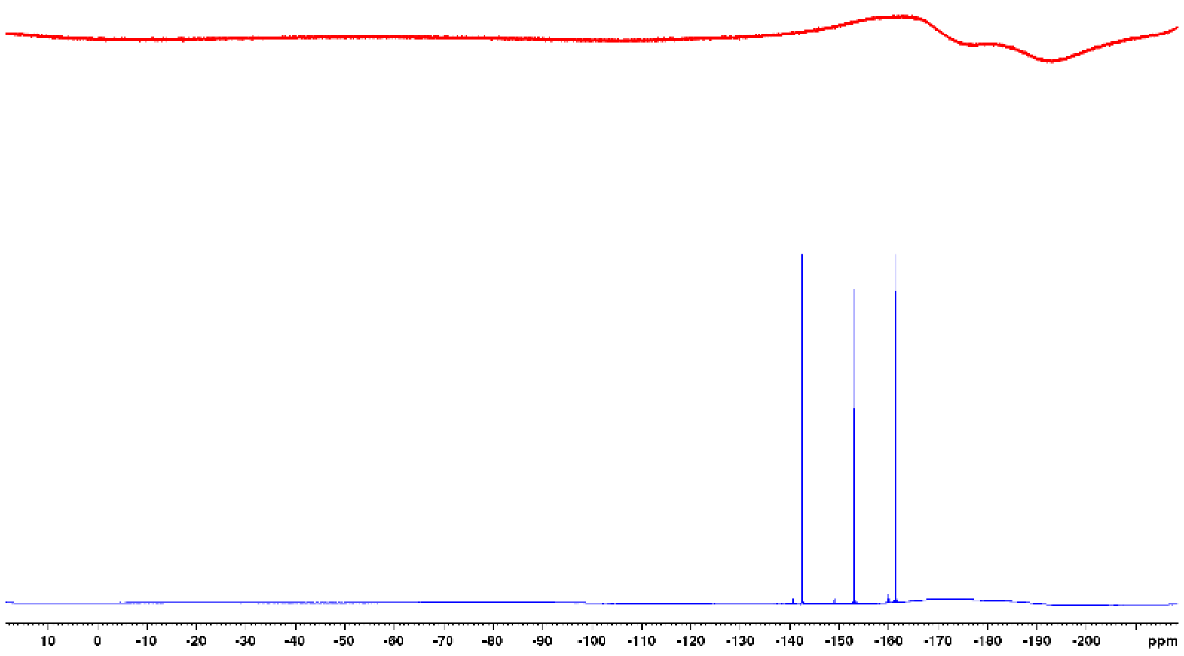

Figure S2. ^{19}F-NMR spectra of Br5FQ (blue) and Br5CzQ (red) synthesized in the study. Recorded in $CDCl_3$.

From the ^{19}F-NMR recorded for the starting Br5FQ, the three peaks of the F atoms can be clearly seen. These peaks are absent from the carbazole substituted Br5CzQ, confirming the successful substitution for Br5CzQ.

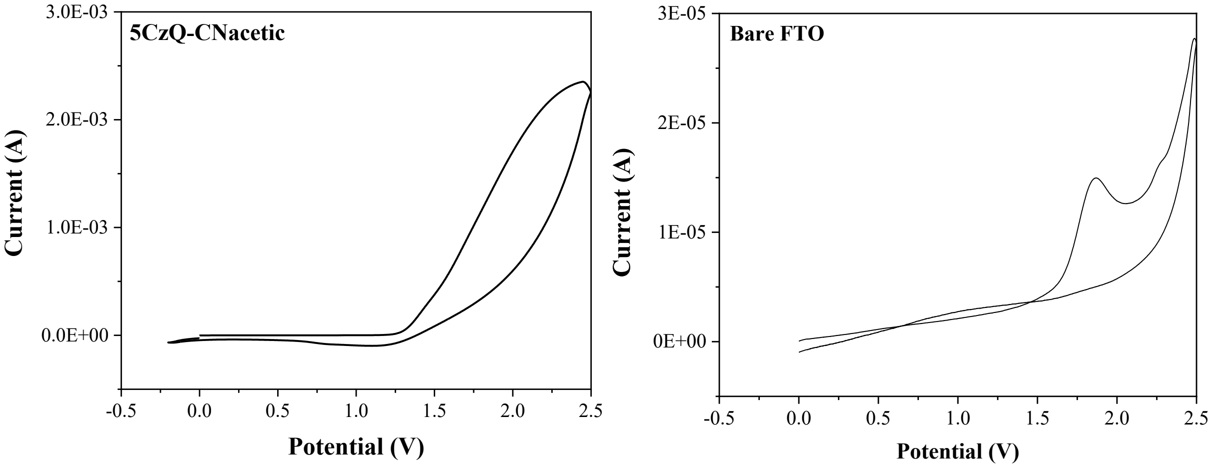

Figure S3. Cyclic voltammogram of the quinoline-based dye (left) and the bare working electrode (right).

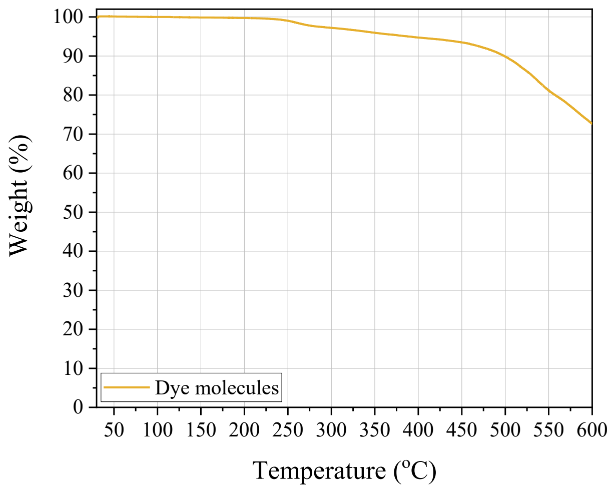

Figure S4. Thermogravimetric analysis (TGA) curve for the quinoline-based dye.
